# Early detection value of miR-29a in patients with acute myocardial infarction

**DOI:** 10.3389/fcvm.2026.1744328

**Published:** 2026-05-11

**Authors:** Yanqing Wang, Peng Gao, Yawei Duan, Hanqi Su, Haibo Wu, Yuan Jia, Yue Zhang, Jia Liu, Hongxiao Li, Xiangming Li, Wenjuan Li, Xiaoshuai Shi, Tingting Liu, Teng Huang, Rongpin Du

**Affiliations:** 1 Department of Internal Medicine, Hebei Medical University, Shijiazhuang, Hebei, China; 2 Departments of Cardiology, Hebei General Hospital, Shijiazhuang, Hebei, China

**Keywords:** acute myocardial infarction, miR-29a, myocardial remodeling, SST2, SYNTAX scores

## Abstract

**Background:**

Early detection of myocardial remodeling, a critical precursor to heart failure in acute myocardial infarction (AMI), remains inadequate with current biomarkers. Preclinical studies suggest miR-29a plays a role in these processes, yet its diagnostic value in early AMI remains unclear. This study evaluates the diagnostic and prognostic potential of miR-29a as a marker for myocardial remodeling in AMI patients.

**Methods:**

A prospective cohort study enrolled 52 patients with ST-elevation myocardial infarction (STEMI) caused by anterior descending branch occlusion, who underwent primary percutaneous coronary intervention within 12 h of symptom onset, along with 39 healthy controls.

**Results:**

Serum miR-29a levels were significantly elevated in STEMI patients compared to controls, exhibiting a graded increase with higher SYNTAX scores. MiR-29a levels positively correlated with soluble suppression of tumorigenicity 2 (sST2), N-terminal pro-brain natriuretic peptide (NT-proBNP), and the Tei index, while showing an inverse correlation with left ventricular ejection fraction (LVEF). Receiver operating characteristic (ROC) analysis indicated that serum miR-29a holds promise as a novel biomarker for early detection of myocardial remodeling in AMI. Furthermore, the combination of miR-29a with NT-proBNP and/or sST2 significantly enhanced diagnostic accuracy compared to individual biomarkers. The triad of miR-29a, NT-proBNP, and sST2 achieved the highest area under the curve (AUC) of 0.904, with 97.4% specificity and 76.9% sensitivity.

**Conclusion:**

Serum miR-29a shows significant potential as a biomarker for the early detection of myocardial remodeling in AMI, providing valuable diagnostic and prognostic insights.

## 1 Introduction

Approximately 25% of patients surviving acute myocardial infarction (AMI) develop heart failure, primarily driven by adverse cardiac remodeling and fibrosis (1, 2). While primary percutaneous coronary intervention (PPCI) remains the cornerstone of AMI management, it inherently induces myocardial ischemia/reperfusion (I/R) injury, initiating a cascade of pathological events including oxidative stress (3, 4), neutrophil infiltration (5, 6), and platelet aggregation (7, 8). Although these processes are part of the initial inflammatory response necessary for tissue repair, they can become maladaptive. The persistence and exacerbation of this inflammatory environment contribute to ongoing tissue damage, creating a substrate that accelerates adverse myocardial remodeling and fibrosis. Numerous studies have sought to identify the primary mechanisms underlying myocardial remodeling and its key biomarkers. Commonly used biomarkers in clinical practice include NT-proBNP (9–11) and high-sensitivity C-reactive protein (hsCRP) (12). Previous studies have demonstrated that admission or peak NT-proBNP levels are strong predictors of both short- and long-term all-cause mortality, cardiovascular mortality, and risk of heart failure readmission in AMI patients. These predictive capabilities remain robust even after accounting for traditional risk factors such as age, renal function, and left ventricular ejection fraction (LVEF). Additionally, dynamic monitoring of NT-proBNP trends often signals poor remodeling and worse clinical outcomes (13, 14). However, these markers fail to accurately reflect the severity of early myocardial remodeling, highlighting the need for better indicators in the early stages of AMI.

Accumulating evidence suggests that soluble suppression of tumorigenicity 2 (sST2), a member of the IL-1 receptor family, plays a significant role in myocardial remodeling. Induced at the transcriptional level by myocardial fibroblasts and cardiomyocytes under stress, sST2 specifically signals pro-fibrotic and pro-inflammatory remodeling processes. Expressed in cardiomyocytes, endothelial cells, and fibroblasts, sST2 has been validated as a biomarker for myocardial remodeling in patients with ischemic cardiomyopathy (15–20). Its secretion increases with myocardial injury and correlates strongly with the progression of myocardial remodeling (21–24). Clinical studies have confirmed that early (e.g., 24 h post-AMI) sST2 levels serve as independent predictors of heart failure progression, with their predictive power remaining significant even after adjustment for age, renal function, LVEF, and NT-proBNP levels (16, 25).

Additionally, research has implicated miR-29a in myocardial remodeling processes (26, 27). Basic studies have shown that activation of the Wnt signaling pathway and upregulation of miR-29a expression contribute to myocardial remodeling and subsequent deterioration of cardiac function (24). However, the potential of miR-29a as a marker for the severity of myocardial remodeling in the early stages of AMI remains unclear. Therefore, this study aims to explore the relationship between miR-29a and myocardial remodeling following AMI, hypothesizing a correlation between serum miR-29a and sST2 levels and comparing their serum relationships.

## 2 Materials and methods

### 2.1 Study population

This study included 52 patients with acute ST-elevation myocardial infarction (STEMI) admitted to Hebei Provincial People's Hospital between June 2022 and July 2023, designated as the STEMI group. These patients were admitted to the emergency department within 12 h of symptom onset and received PPCI. STEMI was diagnosed in accordance with the 2023 ESC Guidelines for the Management of Acute Coronary Syndrome (28). Additionally, 39 healthy volunteers undergoing routine physical examinations during the same period were included as the control group.

Exclusion criteria were as follows: prior diagnosis of coronary artery disease, congenital heart disease, cardiomyopathy, valvular disease, chronic obstructive pulmonary disease, pulmonary interstitial fibrosis, severe hepatic or renal insufficiency, hematological disorders, malignancies, and severe infectious diseases. No significant differences (*P* > 0.05) in general and biochemical data were found between the two groups. The study received approval from the local ethics committee and adhered to the Helsinki Declaration of the World Medical Association (WMA) “Ethical Principles for Medical Research Involving Human Subjects” (updated 2000). Inclusion criteria included participants aged over 18 years who provided written informed consent. The study was registered under the Medical Science Research Project of the Hebei Provincial Health Commission (Project No. 20190279).

### 2.2 Diagnostic workup

At the time of admission, each patient underwent laboratory tests, including clinical chemistry and blood counts. High-sensitivity cardiac troponin (Hs-cTnT) levels were measured upon admission, and all patients received treatment according to the ESC guidelines. miR-29a samples were also collected. Clinical evaluation was performed through history, physical examination, 12-lead electrocardiogram (ECG), continuous ECG monitoring, and pulse oximetry. Additional diagnostic tests were conducted at the attending physician's discretion, including laboratory tests for NT-proBNP and sST2, and echocardiography. Final STEMI diagnosis was confirmed by a cardiologist based on clinical and diagnostic findings.

### 2.3 Echocardiographic study

Standard transthoracic echocardiography (CX50, ROYAL PHILIPS, Netherlands) was performed within the first day following STEMI onset. Data were obtained from parasternal and apical views using a sector matrix probe, with all images captured by an independent ultrasound specialist who was blinded to patient history. The control group underwent the same protocol for echocardiographic assessment at the ultrasound department of the physical examination center. Key echocardiographic parameters included left ventricular end-diastolic volume (LVEDV), left ventricular end-systolic volume (LVESV), left ventricular outflow tract velocity-time integral (LVOT-VTI), Tei index, and LVEF, with the latter evaluated using the Simpson method.

### 2.4 Laboratory measurements

Blood samples were collected 12 h post-admission from STEMI patients. Serum levels of sST2, NT-proBNP, and cTnT were measured using the sST2 detection kit (enzyme-linked immunoassay, C&D Company, USA), the NT-proBNP detection kit (time-resolved immunofluorescence assay, R&M Company, Denmark), and the cTnT test kit (time-resolved immunofluorescence, R&M Company, Denmark), respectively. Experienced laboratory technicians followed the kit instructions strictly for all assays. Blood samples were collected in K2EDTA tubes, centrifuged at room temperature (approximately 22 °C) at 4,000 rpm for 10 min, and plasma was stored at −80 °C for later analysis.

### 2.5 MiR-29a expression

In the research groups, 3 mL of venous blood was collected 12 h post-admission from patients. The blood was collected in vacuum tubes without anticoagulants, centrifuged at a radius of 5 cm and 3,000 rpm for 10 min. The serum supernatant was transferred to RNase-free tubes and stored at −80 °C for subsequent testing. Total RNA was extracted using TRIzol reagent (Ambion, Texas, USA) according to the manufacturer's protocol. RNA concentration was measured using a NanoPhotometer N50 (Impen, Munich, Germany). cDNA synthesis was performed using the SureScript-First-strand-cDNA-synthesis-kit (GeneCopoeia, Maryland, USA) on an S1000^TM^ Thermo Cycler (Bio-Rad, California, USA). Quantitative PCR (qPCR) was carried out using a CFX Connect Real-time Quantitative PCR Instrument (Bio-Rad, California, USA) with the following conditions: pre-denaturation at 95 °C for 1 min, denaturation at 95 °C for 20 s, annealing at 55 °C for 20 s, and extension at 72 °C for 30 s, for a total of 40 cycles. The primers used were as follows: miR-29a, forward 5′-CTGGTGTCGTGGAATTCAGTTGA-3′, reverse 5′-CCTGGCTCCTCACTTGGC-3′; U6, forward 5′-CTCGCTTCGGCAGCACA-3′, reverse 5′-AACGCTTCACGAATTTGCGT-3′. U6 was used as the reference gene for miR-29a. Relative quantification of mRNA expression was calculated using the 2^−ΔΔCt^ method.

### 2.6 Statistical analysis

Statistical analysis was performed using SPSS 26.0 software (IBM, New York, USA). All continuous data are expressed as mean ± standard deviation (x ± s). The comparison of two groups was performed using the *t*-test, while categorical data are presented as percentages, with statistical significance assessed by the chi-square test. For non-normally distributed biomarkers, we applied non-parametric tests. Multivariate logistic regression analysis adjusted for age, sex, and BMI between AMI patients and controls. Linear correlation analysis between miR-29a levels and other biomarkers was conducted using Pearson's method. Receiver operating characteristic (ROC) curve analysis was performed to evaluate the diagnostic value of miR-29a in AMI. Statistical significance was defined as *P* < 0.05, with a significance threshold of *P* < 0.1 for multivariate logistic regression analysis due to the small sample size.

## 3 Results

### 3.1 Baseline characteristics

The characteristics of the study populations are detailed in Table 1. Multivariate logistic regression analysis, adjusted for age, sex, and BMI, alongside miR-29a levels, revealed that both miR-29a and BMI were significant predictors of AMI (miR-29a: OR = 18.264, 95% CI 0.220–1,517.014, *p* < 0.098; BMI: OR = 3,950.530, 95% CI 19.567–658,859.429, *p* = 0.002). Obesity, a well-established risk factor for cardiovascular disease, aligns with the observed pathophysiological pattern, as AMI patients tended to have a higher average BMI.

**Table 1:** Characteristics of the study populations.

Characteristics	STEMI group	Control group	*P*-value
	(*n* = 52)	(*n* = 39)	
Age (Years)	59.04 ± 11.69	51.92 ± 10.57	0.004*
Male [*n* (%)]	46 (88.46)	26 (66.67)	0.011*
Risk factors			
BMI (kg/m^2^)	25.03 ± 0.40	23.67 ± 0.38	0.001*
Smoking [*n* (%)]	26 (50)	19 (48.7)	0.904
Arterial Hypertension [*n* (%)]	29 (55.8)	21 (53.8)	0.855
Diabetes mellitus [*n* (%)]	10 (19.2)	5 (13.2)	0.445
Clinical chemistry			
TC (mmol/L)	4.72 ± 0.98	5.48 ± 1.00	0.001*
TG (mmol/L)	1.43 ± 0.96	2.78 ± 2.40	0.002*
LDL-C (mmol/L)	3.12 ± 0.71	3.35 ± 0.63	0.113
HDL (mmol/L)	1.09 ± 0.22	1.35 ± 0.44	0.001*
Cr (umol/L)	72.6 ± 29.98	71.96 ± 13.13	0.901
GFR (mL/min)	96.53 ± 18.51	101.19 ± 11.36	0.169
UA (umol/L)	350.66 ± 113.00	391.40 ± 114.74	0.096
WBC (×109/L)	10.95 ± 3.40	6.12 ± 1.32	0.001*
NEUT (%)	77.29 ± 13.79	56.32 ± 7.25	0.001*
Drug use (*n*, %)			
*Β*-blockers [*n* (%)]	3 (5.8)	1 (2.6)	0.545
ACEI [*n* (%)]	10 (19.2)	7 (17.9)	0.877
MRA [*n* (%)]	0 (0)	0 (0)	
Vericiguat [*n* (%)]	0 (0)	0 (0)	

BMI, Body Mass Index; TC, Total cholesterol; TG, Triglyceride; LDL-C, Low-density lipoprotein cholesterol-c; HDL, High-density lipoprotein cholesterol; Cr, Creatinine; GFR, Glomerular filtration rate; UA, Uric acid; WBCm White blood cell; NEUT, Neutrophil percentage; ACEI, Angiotensin-converting enzyme inhibitors; MRA, Mineralocorticoid Receptor Antagonist.

### 3.2 Comparison of left ventricular function parameters between the AMI group and the control group

Quantitative echocardiographic assessment demonstrated significant impairment in both left ventricular systolic and diastolic function in the AMI cohort compared to controls, as shown in Table 2. AMI patients exhibited notably reduced LVEF (50.69% ± 8.28% *vs.* 58.49% ± 2.79%; *p* < 0.001), indicating compromised systolic performance. Additionally, a markedly elevated Tei index (0.49 ± 0.09 *vs.* 0.36 ± 0.04; *p* < 0.001) further indicated substantial impairment in global ventricular function. Stroke volume, as assessed by LVOT-VTI, was significantly reduced (17.78 ± 4.98 *vs.* 21.29 ± 3.89; *p* < 0.001), alongside abnormal left ventricular contractility characterized by significantly smaller LVESV (45.35 ± 2.92 *vs.* 52.91 ± 2.19; *p* < 0.001) and LVEDV (108.86 ± 4.70 *vs.* 123.82 ± 5.56; *p* < 0.001), confirming the severe cardiac dysfunction of AMI.

**Table 2:** Comparison of relevant parameters of Two groups of echocardiograms.

Group	*n*	LVEF (%)	Tei index	LVOT-VTI (cm)	LVESV (mL)	LVEDV (mL)
Control group	39	58.4 ± 2.79	0.36 ± 0.04	21.29 ± 3.89	52.91 ± 2.19	123.82 ± 5.56
STEMI group	52	50.69 ± 8.28	0.49 ± 0.09	17.78 ± 4.98	45.35 ± 2.92	108.86 ± 4.70
*P*-value		<0.001	<0.001	<0.001	<0.001	<0.001

LVEF, Left ventricular ejection fraction; LVOT-VTI, Left ventricular outflow tract-velocity time integral; LVESV, Left Ventricular End-Diastolic Volume, LVEDV, Left Ventricular End-Diastolic Volume.

### 3.3 Comparison of cTnT, NT-proBNP, sST2, and miR-29a between the AMI group and the control group

Plasma biomarker analysis revealed significant dysregulation in myocardial injury and cardiac fibrosis markers in AMI patients compared to controls, as detailed in Table 3. cTnT, a specific marker of cardiomyocyte necrosis, was markedly elevated in the AMI group (1,742.96 ± 4,417.57 ng/mL *vs.* 77.92 ± 25.51 ng/mL; *P* = 0.009), although with considerable variability. Additionally, biomarkers reflecting ventricular stress and remodeling showed dramatic alterations: NT-proBNP levels surged 7.6-fold (1,374.48 ± 1,636.97 pg/mL *vs.* 181.26 ± 105.54 pg/mL; *P* < 0.001), while sST2 increased by 61% (24.58 ± 9.67 U/mL *vs.* 15.31 ± 2.78 U/mL; *P* < 0.001). Most notably, miR-29a exhibited a 97.6% overexpression in the AMI cohort (3.26 ± 2.45 *vs.* 1.65 ± 0.37; *P* < 0.001), marking its perturbation as a feature of the post-infarction molecular landscape.

**Table 3:** Comparison of cTnT, NT-proBNP, sST2, and miR-29a-related parameters between the two groups.

Group	*n*	cTnT (ng/mL)	NT-proBNP (pg/mL)^a^	sST2(ng/mL)	miR-29a
Control group	39	77.92 ± 25.51	181.26 ± 105.54	15.31 ± 2.78	1.65 ± 0.37
STEMI group	52	1,742.96 ± 4,417.57	1,374.48 ± 1,636.97	24.58 ± 9.67	3.26 ± 2.45
*P*-value		0.009	<0.001	<0.001	<0.001

cTnT, Cardiac troponin T; NT-proBNP, N-terminal pro-brain natriuretic peptide; sST2, growth STimulation expressed gene 2.

a non-parametric tests.

### 3.4 Comparison of left ventricular function parameters between subgroups in the AMI group and the control group

Stratification of AMI patients by SYNTAX score revealed a graded deterioration in left ventricular function with increasing coronary disease complexity, as shown in Table 4. Compared to controls, all AMI subgroups demonstrated significant functional impairment. Patients with SYNTAX scores ≤22 exhibited moderately reduced systolic function and compromised global performance. Compared to patients with SYNTAX scores ≤22 and 23-32, LVEF (49.76% ± 7.23 *vs.* 53.62% ± 6.65; *p* < 0.05) and Tei index (0.49 ± 0.07 *vs.* 0.46 ± 0.09; *p* < 0.05) showed further worsening. The most severe functional derangement was observed in patients with high SYNTAX scores (≥33), who had markedly depressed LVEF (46.50% ± 11.49% *vs.* 49.76% ± 7.23%; *p* < 0.05) and the highest Tei index (0.55 ± 0.11 *vs.* 0.49 ± 0.07; *p* < 0.05). LVOT-VTI, LVESV, and LVEDV were significantly reduced across all AMI subgroups compared to controls. Notably, despite the disease complexity, left ventricular structure in the early stage of AMI remained in a compensatory state, with no significant changes observed (*p* > 0.05), indicating that these structural parameters do not alter rapidly in the acute phase.

**Table 4:** Comparison of relevant echocardiographic parameters among AMI subgroups and the control group.

Group	*n*	LVEF (%)	Tei index	LVOT-VTI (cm)	LVESV (mL)	LVEDV (mL)
Control group	39	58.49 ± 2.79	0.36 ± 0.04	21.29 ± 3.89	52.91 ± 2.19	123.82 ± 5.56
STEMI group						
SYNTAX score ≤ 22分	21	53.62 ± 6.65*ab	0.46 ± 0.09*ab	18.72 ± 3.42*a	53.59 ± 2.20*	124.10 ± 5.53*
SYNTAX score 23-32分	21	49.76 ± 7.23#c	0.49 ± 0.07#c	16.66 ± 4.97#c	52.16 ± 1.90#	123.05 ± 5.47#
SYNTAX score ≥33分	10	46.50 ± 11.49&	0.55 ± 0.11&	17.76 ± 7.45&	53.06 ± 2.44&	124.85 ± 6.14&

LVEF, Left ventricular ejection fraction; LVOT-VTI, Left ventricular outflow tract-velocity time integral; LVESV, Left Ventricular End-Diastolic Volume; LVEDV, Left Ventricular End-Diastolic Volume.

*P < 0.05: There were significant differences between the control group and SYNTAX score (≤22 points, 23-32 points, ≥33 points) data, as indicated by *, #, and &. *P < 0.05: Among the AMI groups, there were significant differences between the ≤22 group and the (23-32 points, ≥33 points) data, as indicated by a and b.

*P < 0.05: There is a significant difference between the 23-32 group and the ≥33 group, represented by c.

### 3.5 Comparison of cTnT, NT-proBNP, sST2, and miR-29a between subgroups in the AMI group and the control group

Patients with AMI demonstrated significantly altered serum biomarker profiles compared to controls, with levels showing a graded increase that correlated with coronary artery disease complexity as quantified by SYNTAX scores (Table 5). The SYNTAX 23-32 group displayed significantly higher levels of NT-proBNP (1,568.29 ± 1,847.16 pg/mL *vs.* 557.62 ± 831.94 pg/mL, *p* ≤ 0.05) and miR-29a (2.57 ± 1.00 *vs.* 1.98 ± 0.52, *p* ≤ 0.05) compared to the SYNTAX ≤22 group. The SYNTAX ≥33 group showed even greater elevations in NT-proBNP (2,682.90 ± 1,586.71 pg/mL *vs.* 557.62 ± 831.94 pg/mL, *p* ≤ 0.05) compared to the SYNTAX ≤22 group. However, while there was an upward trend in NT-proBNP levels between the SYNTAX ≥33 and SYNTAX 23-32 groups, no significant differential expression was observed, possibly due to the small sample size. Compared to controls, all AMI cohorts exhibited significantly elevated sST2 levels, with the following values: low SYNTAX ≤22 (21.42 ± 9.39 ng/mL), intermediate SYNTAX 23-32 (25.20 ± 9.42 ng/mL), and high SYNTAX ≥33 (29.87 ± 9.13 ng/mL), compared to control levels (15.31 ± 2.78 ng/mL; all *p* < 0.05), confirming extensive myocardial necrosis across all severity strata. The SYNTAX ≥33 group showed significantly higher sST2 levels (29.87 ± 9.13 ng/mL *vs.* 21.42 ± 9.39 ng/mL, *p* ≤ 0.05) compared to the SYNTAX ≤22 group. Notably, miR-29a, a key regulator of extracellular matrix (ECM) remodeling, exhibited a profound, severity-dependent overexpression across SYNTAX groups (low SYNTAX ≤22 [1.98 ± 0.52], intermediate SYNTAX 23-32 [2.57 ± 1.00], high SYNTAX ≥33 [7.34 ± 2.83] *vs.* control [1.65 ± 0.37]; all *p* < 0.05). Moreover, significant increments in miR-29a were observed between each SYNTAX tier, establishing a quantifiable molecular link to angiographic disease complexity.

**Table 5:** Comparison of cTnT, NT-proBNP, sST2, and miR-29a related parameters among AMI subgroups and the control group.

Group	*n*	cTnT (ng/mL)	^NT-proBNP (pg/mL)	ST2(ng/mL)	miR-29a
Control group	39	77.92 ± 25.51	181.26 ± 105.54	15.31 ± 2.78	1.65 ± 0.37
STEMI group					
SYNTAX score ≤22分	21	1,671.57 ± 5,612.97	557.62 ± 831.94	21.42 ± 9.39*	1.98 ± 0.52*
SYNTAX score 23-32分	21	1,246.76 ± 1,877.28#	1,568.29 ± 1,847.16#a	25.20 ± 9.42#	2.57 ± 1.00#a
SYNTAX score ≥33分	10	2,934.90 ± 5,529.56&	2,682.90 ± 1,586.71&b	29.87 ± 9.13&b	7.34 ± 2.83&bc

cTnT, Cardiac troponin T; NT-proBNP, N-terminal pro-brain natriuretic peptide; sST2, growth STimulation expressed gene 2.

*P < 0.05: There were significant differences between the control group and SYNTAX score (≤22 points, 23-32 points, ≥33 points) data, as indicated by *, #, and &. *P < 0.05: Among the AMI groups, there were significant differences between the ≤22 group and the (23-32 points, ≥33 points) data, as indicated by a and b.

*P < 0.05: There is a significant difference between the 23-32 group and the ≥33 group, represented by c.

^non-parametric tests.

### 3.6 The correlation between serum miR-29a levels in the AMI group and the levels of cTnT, NT-proBNP, sST2, LVEF, LVOT-VTI, LVESV, LVEDV, and Tei index

**Figure 1:**
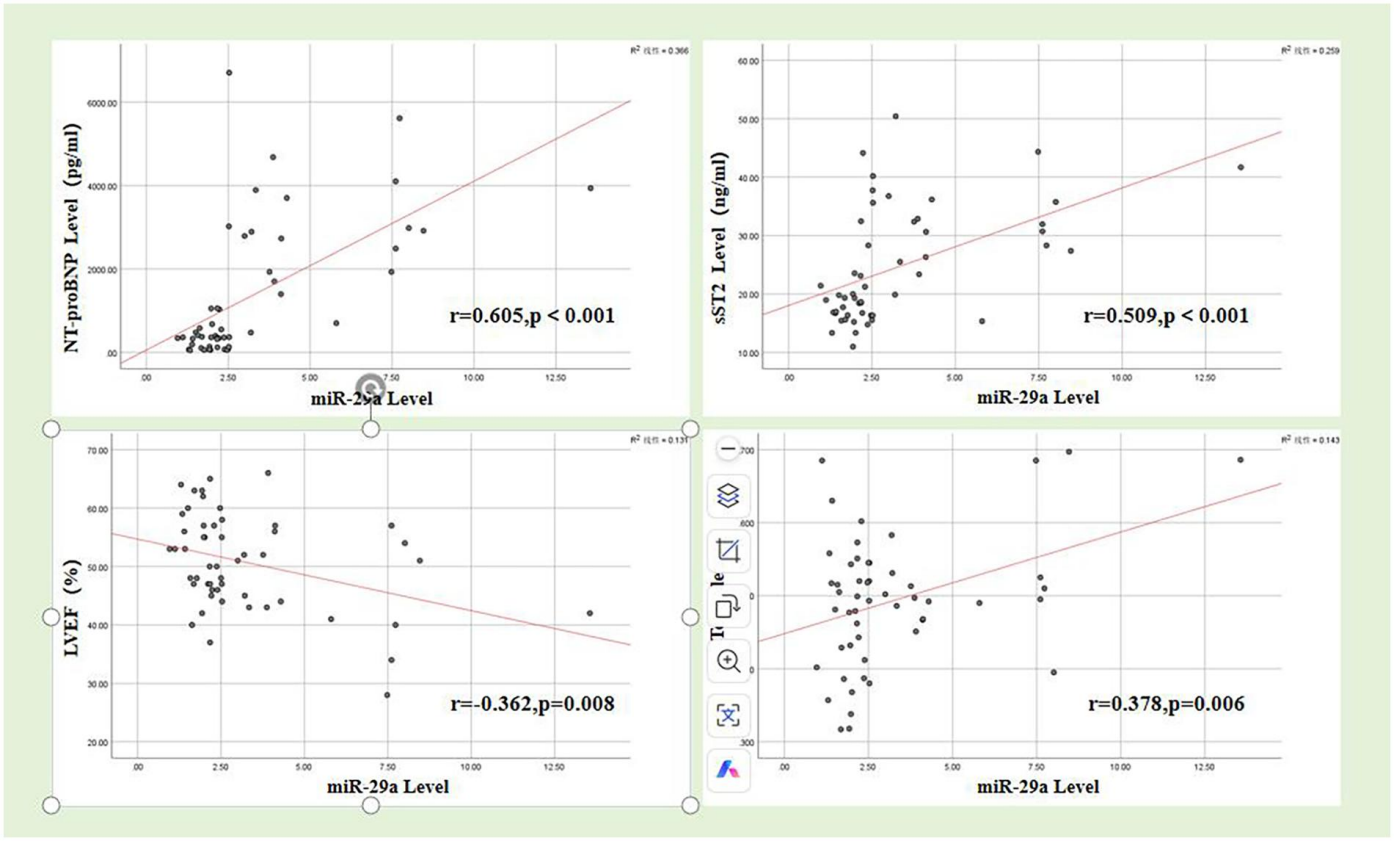
The correlation between serum miR-29a levels in AMI group and the levels of cTnT, NT-proBNP, sST2, LVEF, LVOT-VTI, LVESV, LVEDV and Tei index.

Correlation analysis within the AMI cohort revealed that miR-29a was associated with myocardial injury, cardiac function, and other related indicators (Table 6 and Figure 1). Serum miR-29a levels demonstrated a strong positive correlation with sST2 (*r* = 0.509, *P* < 0.001), highlighting its role in ECM remodeling. Additionally, significant positive associations were observed with NT-proBNP (*r* = 0.605, *P* < 0.001) and Tei index (*r* = 0.378, *P* < 0.006), suggesting its broad relevance across pathophysiological domains. Conversely, miR-29a showed a significant inverse correlation with LVEF (*r* = −0.362, *P* = 0.008). Notably, miR-29a levels were not significantly correlated with cTnT (*r* = 0.225, *P* = 0.109), LVOT-VTI (*r* = −0.052, *P* = 0.716), LVESV (*r* = −0.080, *P* = 0.573), or LVEDV (*r* = 0.134, *P* = 0.344). This distinct correlation profile positions miR-29a as a potential molecular indicator for myocardial remodeling in AMI.

**Table 6:** Correlation between serum miR-29a levels in the AMI group and the levels of cTnT, NT-proBNP, sST2, as well as LVEF, LVOT-VTI, LVESV, LVEDV, and Tei index.

Statistics	cTnT	NT-proBNP	sST2	LVEF	LVOT-VTI	LVESV	LVEDV	Tei index
*r*	0.225	0.605	0.509	−0.362	−0.052	−0.080	0.134	0.378
*P*	0.109	<0.001	<0.001	0.008	0.716	0.573	0.344	0.006
*n*	52	52	52	52	52	52	52	52

Pearson correlation analysis showed that in the AMI group, serum miR-29a levels were positively correlated with NT-proBNP, sST2 levels, and Tei index (*r* = 0.605, 0.509, 0.378, *P* < 0.05), and negatively correlated with LVEF (*r* = −0.362, *P* = 0.008). There was no correlation with LVOT-VTI, LVESV, or LVEDV values, with *P* values of 0.716, 0.573, and 0.344, respectively.

### 3.7 The ROC curve of miR-29a for diagnosing heart failure after AMI

**Figure 2:**
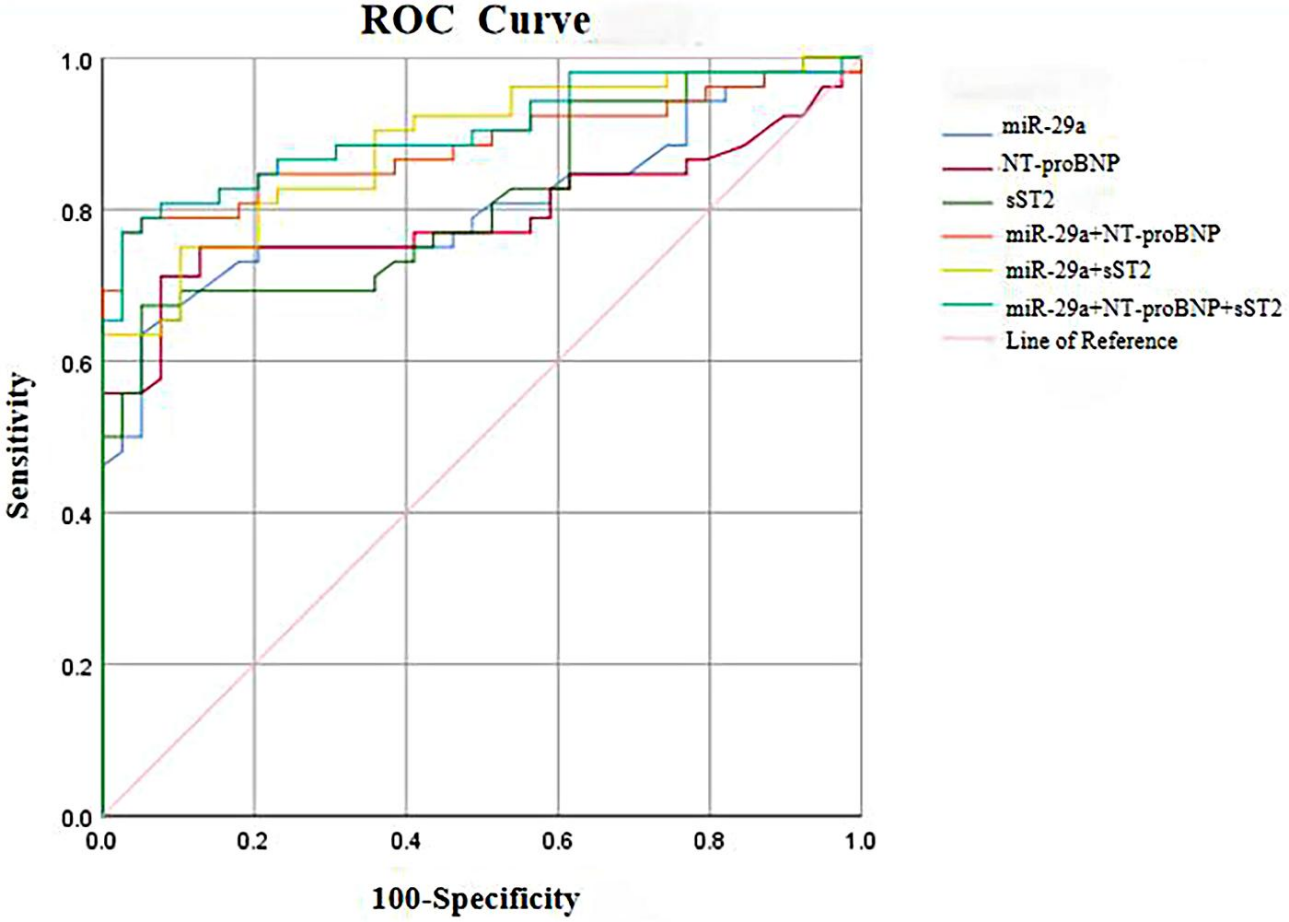
The ROC curve of serum miR-29a, NT-proBNP, sST2 and miR-29a + NT-proBNP, miR-29a + sST2, miR-29a + NT-proBNP + sST2 for diagnosing heart failure after AMI.

ROC curve analysis was performed for serum miR-29a, NT-proBNP, sST2, and their combinations (Figure 2). The area under the ROC curve (AUC) for each biomarker was as follows: miR-29a alone (AUC = 0.803,95% CI 0.711–0.894, *p* < 0.001), NT-proBNP alone (AUC = 0.792,95% CI 0.695–0.889, *p* < 0.001), sST2 alone (AUC = 0.812,95% CI 0.725–0.899, *p* < 0.001), miR-29a + NT-proBNP (AUC = 0.883, 0.811–0.956, *p* < 0.001), miR-29a + sST2 (AUC = 0.884,95% CI 0.817–0.951, *p* < 0.001), and miR-29a + NT-proBNP  + sST2 (AUC = 0.904,95% CI 0.841–0.967, *p* < 0.001). Notably, combining miR-29a with NT-proBNP (specificity 0.974, sensitivity 0.769) or sST2 (specificity 0.897, sensitivity 0.750) significantly improved diagnostic performance. The triad of miR-29a, NT-proBNP, and sST2 (specificity 0.974, sensitivity 0.743) further enhanced diagnostic performance, as evidenced by the higher AUC and Youden index.

## 4 Discussion

Pathological myocardial remodeling following AMI is a critical factor influencing long-term patient survival, with myocardial fibrosis, characterized by collagen deposition, playing a pivotal role in this process (29–33). Numerous studies have demonstrated that the severity of myocardial fibrosis after AMI serves as an independent risk factor for heart failure and cardiac mortality (34, 35). Accurately monitoring dynamic changes in myocardial fibrosis is essential not only for understanding disease progression mechanisms but also for risk stratification, guiding personalized interventions, and improving patient prognosis (36). Currently, sST2 is widely used in clinical practice to assess the burden of myocardial fibrosis and predict adverse outcomes in AMI patients (37–39). However, sST2 expression is influenced by systemic inflammation, limiting its ability to specifically reflect early ECM metabolic disturbances (40–43). Recent research has highlighted the critical role of miRNAs in the regulatory network of myocardial fibrosis (24, 44). Among these, miR-29a, abundantly expressed in the myocardium, is involved in both myocardial fibrosis and reperfusion injury in AMI, promoting the progression of myocardial remodeling (45–47). This study identified a positive correlation between miR-29a expression and the severity of coronary artery disease in AMI patients, positioning miR-29a as a promising early biomarker for assessing ECM imbalance and fibrosis after AMI.

This study is the first to evaluate the expression profile and combined predictive value of serum miR-29a in AMI through a case-control design. In AMI patients, miR-29a was significantly upregulated compared to the control group (3.26 ± 2.45 *vs.* 1.65 ± 0.37, *P* < 0.001), and a strong positive correlation was found with sST2 (*r* = 0.509, *p* < 0.001). These findings suggest that miR-29a may play a pivotal role as a regulator of early ECM remodeling, aligning with previous studies that identify miR-29a as a key molecule in ECM regulation. Clinical observations further support that miR-29a reflects the pathological response of myocardial stress within the first 24 h after PPCI. Raquel Del Toro et al. also reported the differential expression of 1,209 miRNAs in AMI patients 6 h after PPCI, with a significant increase in miR-29a confirmed by RT-qPCR. Similarly, their ROC analysis demonstrated high specificity and sensitivity, consistent with our findings (48). Additionally, Isabel Galeano Otero et al. observed differential expression of miR-29a prior to PPCI in STEMI patients, although their study did not explicitly associate miR-29a with left ventricular abnormal remodeling (LVAR) (49). In contrast, our study reveals that miR-29a not only correlates strongly with sST2 but also with NT-proBNP levels and the Tei index, while negatively correlating with LVEF. However, during the early phase of AMI, miR-29a does not correlate with LVEDV/LVESV or cTnT. This selective pattern of correlation suggests that miR-29a may differ from cTnT and NT-proBNP—both of which reflect myocardial pathophysiological status—and may specifically relate to left ventricular remodeling.

Strong evidence suggests that in the context of ischemia-reperfusion injury, the release of inflammatory mediators from myocardial cells undergoing cell death contributes to inflammation (50). The inflammatory response plays a critical role in AMI, influencing infarct size and subsequent left ventricular remodeling (51, 52). Additionally, under I/R conditions, myocardial cells release miRNAs associated with extracellular vesicles, indicating that stress factors can trigger the release of extracellular miRNAs involved in remodeling, such as miR-29a (53). Several studies have highlighted the involvement of miR-29a in the inflammatory processes of atherosclerosis (54) and its relation to left ventricular remodeling following AMI (55, 56). Further, basic research shows that miR-29a overexpression can prevent apoptosis and fibrosis gene expression changes induced by ischemia-reperfusion in rat models (57). Numerous studies have demonstrated that miR-29a is highly expressed in the early stages of AMI and is associated with both inflammatory response and abnormal left ventricular remodeling (58–60). The correlation between abnormal left ventricular remodeling and the complexity of coronary artery disease has not been extensively analyzed in previous clinical trials. However, this study confirms that miR-29a expression is significantly elevated, being 3.7 times higher in the SYNTAX ≥33 group compared to the low-risk group (7.34 ± 2.83 *vs.* 1.98 ± 0.52, *p* < 0.001). As the complexity of AMI increases, miR-29a shows a graded ability to warn of the severity of myocardial remodeling. Moreover, although no significant correlation was found between miR-29a and volume indices (LVESV/LVEDV), a strong correlation was observed with LVEF and the Tei index, indirectly confirming that miR-29a is primarily involved in early molecular-level left ventricular remodeling. In addition, echocardiographic parameters obtained during the acute phase may reflect a combination of ischemia, stunning, and loading, indicating early remodeling rather than fixed structural changes. Future studies with serial imaging at 3–6 months will be necessary to confirm the relationship between miR-29a levels and chronic ventricular remodeling.

This study used ROC curve analysis to evaluate the diagnostic value of NT-proBNP, sST2, and miR-29a in AMI. The results showed that the combined use of miR-29a + NT-proBNP or miR-29a + sST2 was more effective in diagnosing AMI than any of these biomarkers alone. The three-biomarker combination of miR-29a + sST2 + NT-proBNP showed further improvement, with a specificity of 0.974 and a sensitivity of 0.750. This combined diagnostic model integrates the pathological and physiological processes of myocardial necrosis and fibrosis in the necrotic area post-AMI, providing a comprehensive evaluation approach for the early identification of high-risk populations for heart failure. However, this study still needs to verify the association between miR-29a and long-term prognosis through a larger sample size, including measures such as 6-month changes in LVEF and heart failure hospitalization rates. Future follow-up studies will be essential to more effectively validate the diagnostic value of miR-29a.

## 5 Limitations

To ensure the robustness of our findings, a formal sample size calculation was conducted prior to the study's initiation. With a two-sided significance level (*α*) of 0.05 and a power (1 − *β*) of 80%, this study estimated that a minimum of 52 AMI patients would be required to detect a clinically meaningful difference in miR-29a levels related to myocardial remodeling and fibrosis. The final cohort included 52 participants, fulfilling the calculated sample size requirement and providing sufficient statistical power for the primary analysis. *post-hoc* power analysis confirmed that the achieved power for detecting the observed effect size in the primary outcome exceeded expectations, further reinforcing the reliability of the reported results. However, as noted in the Limitations section, the sample size may still be insufficient for subgroup analyses or secondary endpoints with smaller effect sizes. These considerations enhance the interpretability of our findings while highlighting the need for validation in larger, multi-center studies. The modest sample size, particularly within subgroups, limits the robustness of distributional assumptions, despite our use of appropriate statistical tests. And while now standardized, the single 12-hour time point may not capture the full kinetic profile of all biomarkers, such as sST2, NT-proBNP and miR-29a.

Other limitations of this study should be acknowledged. Firstly, its cross-sectional design inherently precludes the establishment of causal relationships or definitive prognostic conclusions regarding the observed associations. Secondly, the use of healthy volunteers, rather than patients with non-AMI cardiac conditions, as the control group limits the specificity of the findings for AMI pathophysiology, as the biomarker alterations could be influenced by broader cardiac stress. Third, the absence of serial echocardiographic follow-up data restricts our ability to correlate baseline biomarker levels with the subsequent progression of ventricular remodeling or fibrosis. Finally, the correlation analyses presented are bivariate and lack adjustment for potential confounding variables; therefore, the independent relationship between miR-29a and other parameters requires confirmation through future multivariable analyses in larger cohorts.

## 6 Conclusion

This study suggests that miR-29a may serve as a novel biomarker for the early diagnosis of myocardial remodeling in STEMI patients. Its combined use with NT-proBNP and sST2 offers diagnostic advantages, thereby improving the potential for personalized management following AMI.

## Data Availability

The raw data supporting the conclusions of this article will be made available by the authors, without undue reservation.
